# Spire1 and Myosin Vc promote Ca^2+^-evoked externalization of von Willebrand factor in endothelial cells

**DOI:** 10.1007/s00018-021-04108-x

**Published:** 2022-01-27

**Authors:** Anna Holthenrich, Julian Terglane, Johannes Naß, Magdalena Mietkowska, Eugen Kerkhoff, Volker Gerke

**Affiliations:** 1 Institute of Medical Biochemistry, Center for Molecular Biology of Inflammation, University of Münster, Von-Esmarch-Str. 56, 48149 Münster, Germany; 2 Institute of Molecular Cell Biology, Zoological Institute, Technical University of Braunschweig, Braunschweig, Germany; 3 Department of Neurology, Molecular Cell Biology Laboratory, University Hospital Regensburg, Regensburg, Germany

**Keywords:** Weibel–Palade bodies, Exocytosis, Endothelial activation, Calcium signaling, Actin nucleation

## Abstract

Weibel–Palade bodies (WPB) are endothelial cell-specific storage granules that regulate vascular hemostasis by releasing the platelet adhesion receptor von Willebrand factor (VWF) following stimulation. Fusion of WPB with the plasma membrane is accompanied by the formation of actin rings or coats that support the expulsion of large multimeric VWF fibers. However, factor(s) organizing these actin ring structures have remained elusive. We now identify the actin-binding proteins Spire1 and Myosin Vc (MyoVc) as cytosolic factors that associate with WPB and are involved in actin ring formation at WPB-plasma membrane fusion sites. We show that both, Spire1 and MyoVc localize only to mature WPB and that upon Ca^2+^ evoked exocytosis of WPB, Spire1 and MyoVc together with F-actin concentrate in ring-like structures at the fusion sites. Depletion of Spire1 or MyoVc reduces the number of these actin rings and decreases the amount of VWF externalized to the cell surface after histamine stimulation.

**Supplementary Information:**

The online version contains supplementary material available at 10.1007/s00018-021-04108-x.

## Introduction

Vascular endothelial cells contain unique secretory organelles, the Weibel–Palade bodies (WPB), which store the adhesion receptors P-selectin and von Willebrand factor (VWF) and respond to vascular injury and infection by regulated exocytosis. The rapid release of WPB cargo into the blood is an essential event regulating hemostasis and inflammation, and impaired WPB function is linked to a variety of diseases including von Willebrand disease, the most common inherited bleeding disorder, which is defined by a lack of functional VWF that regulates thrombus formation during hemostasis [1].

WPB form at the trans-Golgi network (TGN) in a process driven by the main cargo VWF [2, 3]. Their subsequent maturation is accompanied by a reduction in pH which facilitates extensive multimerization of VWF leading to the elongated tubular shape of WPB [4, 5]. At the same time, different cytosolic regulators are recruited to WPB. Among them Rab27a was first found associated with mature WPB in 2003 [6] and was shown to facilitate the recruitment of several effectors including MyRIP [7–9] and synaptotagmin-like protein 4 a (Slp4-a) [7]. Different isoforms of Rab3—a classical exocytic Rab GTPase [10]—were also found to localize to WPB [7, 11, 12], although only endogenous Rab3b seems to associate with a large fraction of mature WPB [7]. The exocytotic machinery driving fusion of WPB with the plasma membrane (PM) consists of a plethora of proteins and seems to depend on the type of secretagogue stimulus. Factors involved in facilitating stimulated exocytosis are among others, soluble NSF receptor (SNARE) proteins of the vesicle-associated membrane protein (VAMP) family, VAMP8 and 3 [13, 14], priming factors Munc13-4 and 2 [12, 15, 16], t-SNAREs syntaxin-4 and SNAP23 [13, 14] and the SNARE regulator Munc18-1 and synaptotagmin-5 [17–19].

As an abundant and central protein in many cellular processes, actin has been implicated in several steps during regulation of stimulated secretion. In endothelial cells, actin participates in the short-range movement of WPB [20] and an interaction with polymerized F-actin limits stimulated secretion of WPB, thus facilitating WPB maturation. Specifically, Rab27a and its effector, the myosin and actin-binding protein MyRIP, are recruited to mature WPB and mediate an anchorage of WPB to the actin cortex which prevents exocytosis of not fully matured VWF [8, 21]. This anchorage is supported by the unconventional myosin Va (MyoVa), which binds both actin and MyRIP [8, 9]. Furthermore, actin accumulates in ring or coat-like structures at sites where stimulated PM-WPB fusion has occurred [22–25]. It was proposed that these actin structures stabilize the WPB-associated fusion complex and/or—in conjunction with the conventional mysoin II—actively support the expulsion of large WPB cargo such as highly multimeric VWF [25]. However, despite increasing evidence for the active role of these actin structures in driving the release of highly multimeric VWF, it is not known which factor(s) triggers the recruitment and site-specific polymerization of actin at WPB that have fused with the plasma membrane.

To identify such factor(s), we screened for actin-binding proteins that might associate with WPB. An ascorbate peroxidase 2 (APEX2)-dependent proximity proteomics assay in HUVEC using WPB-associated Rab3b and Rab27a as bait identified the actin-binding proteins MyoVc and Spire1 as possible candidates [16]. Both are of particular interest as actin polymerization-nucleating Spire proteins have been shown to interact directly with proteins of the MyoV family [26] and examples of Rab–Spire–MyoV complexes facilitating the trafficking of different organelles exist [27, 28]. Here we show that both Spire1 and MyoVc localize exclusively to mature WPB in a manner dependent on direct interaction with Rab GTPases. Pull-down experiments suggest an interaction between MyoVc and Spire1 and following histamine stimulation, both proteins localize to ring-like structures at WPB-PM fusion sites. These structures are reminiscent of the post-fusion actin rings described earlier [24] and knockdown of Spire1 or MyoVc reduces the number of such actin rings in conjunction with a decrease in evoked presentation of VWF on the endothelial cell surface.

## Results

### Spire1 and MyoVc localize to mature WPB in a manner dependent on interaction with Rab GTPases

**Fig. 1:**
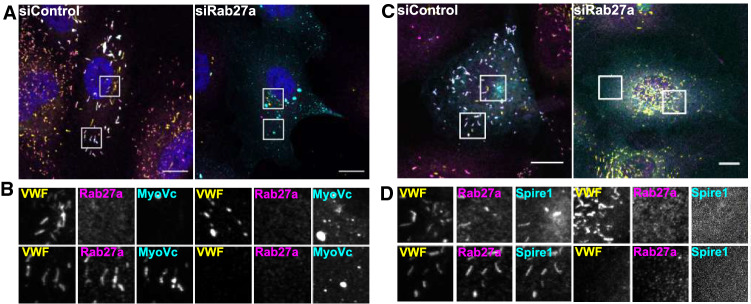
MyoVc and Spire1 localize exclusively to mature WPB. HUVEC were transfected with un-targeting siRNA (siControl) or siRNA directed against Rab27a and MyoVc-GFP or Spire1-GFP (cyan) as indicated. Cells were fixed and stained for VWF as general WPB marker (yellow) or Rab27a as marker for mature WPB using the respective antibodies (magenta). **A** Shows merged pictures of MyoVc-GFP-expressing cells with peri-nuclear and peripheral regions marked by boxes. Boxed regions are enlarged in **B**. DAPI stainings are shown in blue. Scale bars: 10 µm. **C** Shows cells expressing Spire1-GFP. Peri-nuclear and peripheral regions are marked by boxes. Boxed regions are enlarged in **D**. Scale bars: 10 µm

Spire1 and MyoVc are actin-binding proteins that both have been identified as part of the WPB-associated proteome [16]. To confirm the localization to WPB, we overexpressed GFP-tagged versions of both proteins in HUVEC. Interestingly, we observed a clear co-localization with elongated Rab27a-positive WPB, while less elongated and more peri-nuclear WPB positive for VWF but not containing Rab27a lacked any GFP signal (Fig. 1). To corroborate this specific association with mature WPB, we depleted HUVEC of Rab27a, a manipulation which had been shown previously to cause an accumulation of WPB in the peri-nuclear region that fail to undergo full maturation [6, 8, 9]. Figure 1 shows that these peri-nuclear immature WPB do not recruit Spire1 or MyoVc indicating that both proteins are found exclusively on mature WPB.

**Fig. 2:**
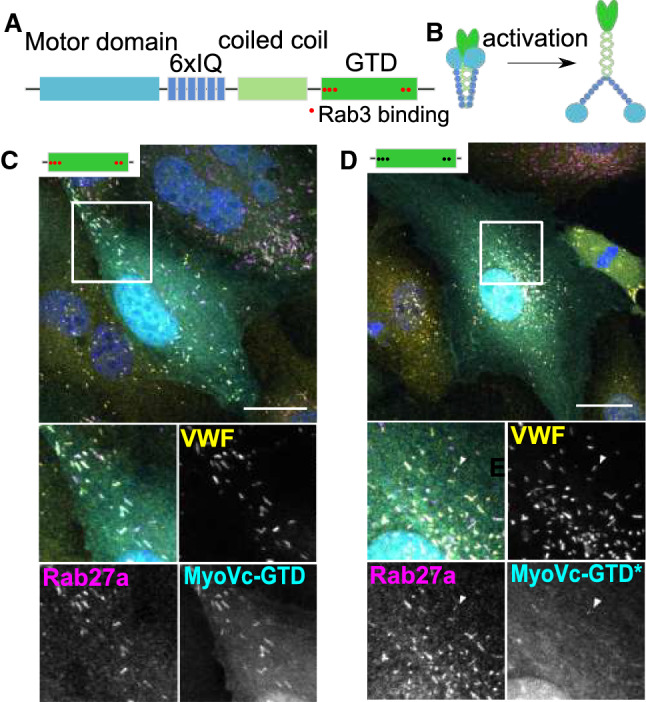
MyoVc localization to WPB is regulated by interaction with Rab3. **A** Depicts the domain structure of MyoVc consisting of a motor domain, 6 IQ domains, a coiled coil domain that drives dimerization, and a globular tail domain (GTD) which facilitates binding to Rab3. **B** Cartoon illustrating that MyoVc forms dimers that unfold upon (cargo-dependent) activation. **C**, **D** HUVEC expressing different GFP-tagged MyoVc mutants (cyan) were co-stained for VWF (yellow) and Rab27a (magenta). DAPI stainings are shown in blue. Scale bars: 10 µm. The boxed areas are shown enlarged beneath the merge. **C** MyoVc-GTD. **D** MyoVc-GTD* mutant deficient in Rab3-binding. The arrowhead depicts a WPB weakly stained for MyoVc-GTD*

The analysis of the WPB-associated proteome had revealed a significant association between MyoVc and Rab3b, another Rab found on mature WPB [16] and MyoVc had been found to directly interact with Rab3 isoforms [29]. Since the globular tail domain (GTD) of MyoVc (cartoon of the MyoVc domain structure and its activation is shown in Fig. 2A, B) facilitates the interaction with Rab3, we expressed GFP-labeled MyoVc-GTD in HUVEC and observed a co-localization with VWF (Fig. 2C). Moreover, MyoVc-GTD-GFP also co-localizes with Rab3b-mApple on VWF-positive WPB (Fig. S1). Considering that the GTD has several binding partners—including a possible interaction with Spire1—we further mutated the five amino acids within the GTD that had been predicted to drive association with Rab3 [29]. This mutation caused a significant loss of the MyoVcGTD*-GFP signal at WPB, although at some WPB-associated MyoVcGTD*-GFP could still be weakly detected (Fig. 2D). This could hint at a possible involvement of a different MyoVc interaction partner in supporting WPB association or at a decreased but not fully abolished binding affinity of the GTD to WPB-associated Rab3.

**Fig. 3:**
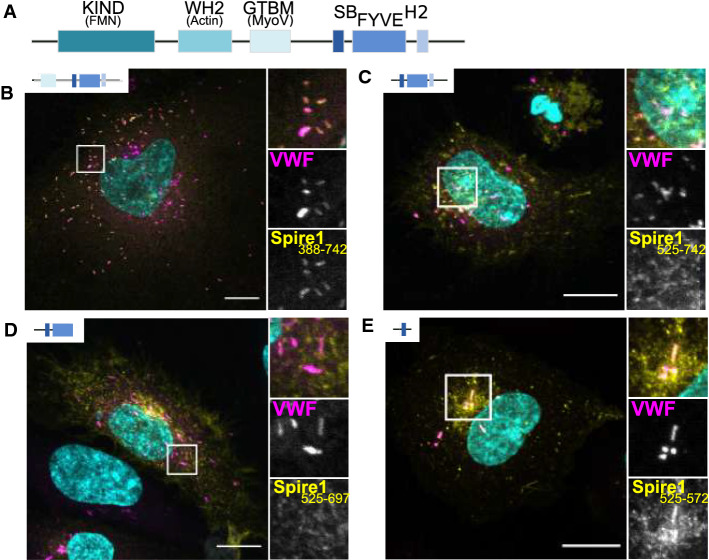
The SB domain of Spire1 is sufficient for mediating recruitment to WPB. **A** Depicts the domain structure of Spire 1 consisting of an actin-binding (WH2) and formin recruiting (KIND) N-terminal part. The C-terminal part comprises a Spire box domain (SB), a FYVE-type zinc finger and a H2 sequence similar to Slp/Slac proteins. **B**–**E** HUVEC were transfected with different GFP-tagged Spire1 truncation mutants (yellow) and co-stained for VWF (magenta). DAPI stainings are shown in cyan. Scale bars: 10 µm. The boxed areas are shown enlarged as a merge (upper picture) and as single channels (lower pictures, VWF and Spire mutants) next to the overview picture showing the whole cell. **B** Spire1 amino acid (aa) 388–742 (GTBM-SB-FYVE-H2, Spire1Tail). **C** Spire1 aa 525–742 (SB-FYVE-H2). **D** Spire1 aa 525–697 (SB-FYVE). **E** Spire1 aa 525–572 (SB)

In mouse oocytes, Spire1 and 2 were shown to function in actin/myosin-dependent Rab11 vesicle transport processes, where the myosin Vb motor protein drives the motility of Rab11 vesicles along a network of actin filaments [28]. The actin filament meshwork is generated by a cooperation of Spire1, 2 and Formin-2 actin nucleators [30]. Protein interaction studies indicate that the GTD of the MyoVb motor protein links the Rab11 GTPase and the Spire actin nucleator into a tripartite complex [26] and Rab11 and Spire proteins were found to interact with distinct sequences of the MyoVb GTD. The vesicular localization of Spire proteins, on the other hand, is mediated by a C-terminal modified FYVE zinc finger motif and flanking sequences [31]. This C-terminal Spire part exhibits high similarity to the Rab-binding domain of the rabphilin 3A protein, which is an effector of Rab GTPases of the secretory group (Rab3, Rab8 and Rab27) [27, 32, 33], and direct binding experiments confirmed an interaction between the Spire1 C-terminal domain and Rab3a [34]. Recently, Alzahofi et al. also reported a direct interaction between Spire1 and Rab27a. Binding of Spire1 to Rab GTPases seems to be facilitated by the Spire Box (SB) domain, which is a conserved motif N-terminally adjacent to the modified FYVE zinc finger and possesses structural similarities to other Rab GTPase-binding domains [27]. Based on these observations, we next assessed the structural basis of the WPB recruitment of Spire1 and a possible involvement of Rab GTPases and, therefore, expressed different GFP-tagged Spire1 truncation mutants in HUVEC. These experiments revealed that the C-terminal part of Spire1 containing the SB was essential for recruitment to the VWF-positive WPB and that the SB domain alone was sufficient for WPB localization (Fig. 3), although the WPB enrichment seemed to be less pronounced in this case. We therefore conclude that Spire1 localization to WPB is driven by interaction with Rab GTPases, most likely Rab27 and Rab3.

As the SB module is well conserved among Spire proteins and Spire2 is also expressed in HUVEC, we analyzed a possible co-localization of GFP-tagged Spire2 with the WPB marker VWF. However, we were unable to identify any association of Spire2 with WPB (Fig. S2).

### Spire1 interacts with MyoVc in HUVEC

**Fig. 4:**
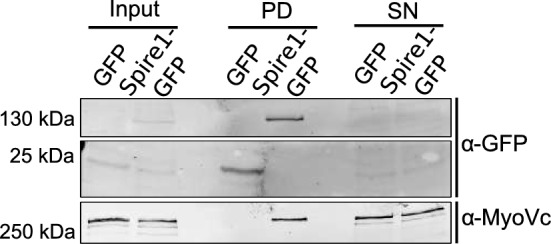
Spire1 interacts with MyoVc. Spire1-GFP or an empty GFP vector (GFP) were expressed ectopically in HUVEC. Cell lysates were directly analyzed by Western blot (Input) or further processed using GFP-trap pull-downs. GFP-trap-bound proteins (pull-down, PD) and the supernatant (SN) of the GFP-trap samples were also analyzed by Western blots using antibodies directed against GFP or MyoVc as indicated

Tripartite complexes between a Spire protein, a Rab GTPase and a Myosin V motor have been observed in various cell types and on different organelles linking de-novo actin nucleation to vesicular transport [35]. While Spire1 was shown to directly interact with MyoVa in different cell types [26, 28], to our knowledge, a possible MyoVc–Spire1 interaction has not been reported. To address this, we expressed Spire1-GFP in HUVEC and pulled down Spire1-GFP and possible interaction partners using a GFP-Trap. Endogenous MyoVc was enriched in the pull-down samples as compared to control cells transfected with an empty GFP vector suggesting that the two proteins can associate with one another (Fig. 4). Pull-down experiments with Spire1 mutants revealed that a region downstream of the KIND and WH2 domains is required for MyoVc binding, which includes the conserved Spire GTBM sequences (Fig. S3). A Spire1 deletion mutant encoding only the SB and FYVE domains did not interact with MyoVc (Fig. S3). This is in line with previous findings mapping the MyoVa/b-binding sites to the central Spire1 GTMB sequences [26].

As MyoVa plays a role in WPB maturation and peripheral anchorage of WPB [9] and the Spire1–Rab27a–MyoVa complex drives dispersion of melanosomes in melanocytes [27], we assessed next whether MyoVc or Spire1 affect the distribution of WPB in endothelial cells. To achieve this, we depleted MyoVc and Spire1 by siRNA-mediated knockdown (Fig. S4) and also expressed GFP-tagged MyoVc or Spire1 full length as well as a MyoVcTail mutant lacking the actin-binding site [36, 37] and a Spire1Tail construct (GTBM-SB-FYVE-H2) depleted of the KIND and WH2 domains, which could act in a dominant-negative manner. As a readout for the (normal) peripheral dispersion of WPB, we assessed whether depletion or overexpression of the MyoVc or Spire1 constructs affects the mean WPB distance to the nucleus. However, none of the treatments had a significant impact on the peripheral distribution of WPB (Fig. S5). The lack of an effect on WPB distribution is unlikely to be caused by a potential compensatory up-regulation of MyoVa in the MyoVc-depleted cells that had been reported before in melanocytes [36], because MyoVa mRNA levels were not altered in our MyoVc-depleted HUVEC (Fig. S4B).

Rab3d, a potential interaction partner of MyoVc, had been linked to WPB biogenesis because overexpression of a dominant-negative Rab3d mutant in HUVEC reduces the number of WPB [11]. Interestingly, we could also observe a slight but significant reduction in the number of WPB per cell after MyoVc knockdown or overexpression of the MyoVcTail construct, a phenotype not seen upon depletion or overexpression of Spire1 constructs (Fig. S6).

### MyoVc and Spire1 promote surface presentation of VWF after histamine stimulation

**Fig. 5:**
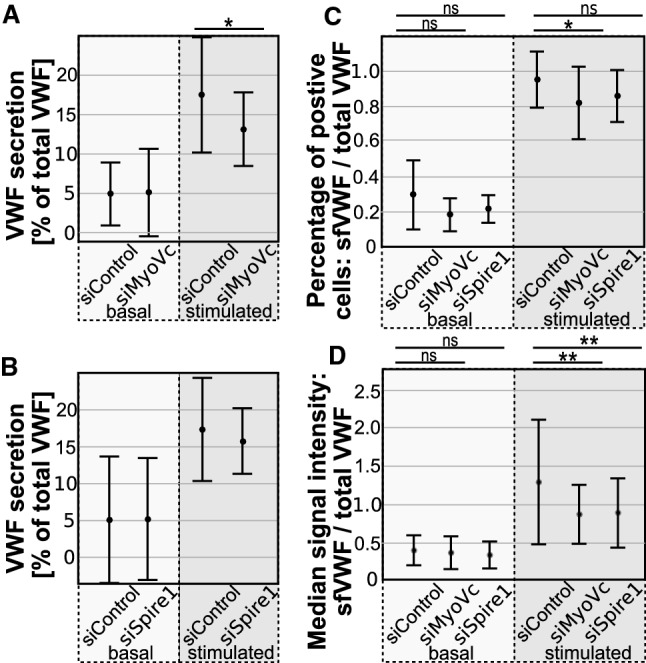
Spire1 and MyoVc positively affect histamine-evoked VWF surface presentation. HUVEC were transfected with siRNA (Control, MyoVc or Spire1) and VWF secretion was measured the following day. **A**,**B** Release of VWF into the culture medium was determined via an ELISA-based assay under resting conditions (basal) and after histamine stimulation (100 µM). Values were correlated to the total amount of VWF which consists of the amount of secreted VWF (basal + stimulated VWF release) and residual VWF (remaining cellular VWF measured in the cell lysates). Statistics were performed using Student’s *t* test. **A** Cells were depleted of MyoVc (siMyoVc). *n* = 6. **B** Cells were depleted of Spire1 (siSpire1). *n* = 5. **C**, **D** Flow cytometry assay employing a Dylight650-conjugated antibody directed against VWF. Antibody capture was carried out on confluent monolayers for 20 min either without (basal) or after the addition of 100 µM histamine (stimulated). Cells were subsequently harvested, stained for total VWF and analyzed via flow cytometry. Shown are values measured for surface VWF (sfVWF) correlated to the total amount of VWF. **C** Percentage of cells positive for surface VWF divided by percentage of cells positive for total VWF. **D** Ratio of the median signal intensity of the surface VWF signal of sfVWF positive cells to the median intensity of the total VWF signal. *n* = 8. Statistics employed one-way ANOVA and Bonferroni-corrected post hoc test. ****p ≤ 0.0001, ***p ≤ 0.001, **p ≤ 0.01, *p ≤ 0.05

To assess whether MyoVc or Spire1 actively participate in the Ca^2+^-regulated exocytosis of WPB, we used an ELISA-based assay to quantify VWF release into the culture medium after stimulation with 100 µM histamine. To this end, we depleted HUVEC of MyoVc or Spire1 and related the amount of released VWF to the total VWF content to correct for effects on WPB formation. We observed a slight decrease of stimulated VWF release into the medium in MyoVc but not Spire1 depleted cells (Fig. 5A, B).

Recent studies suggest that WPB can undergo at least two distinct modes of VWF release after Ca^2+^-evoked exocytosis in cultured HUVEC. A considerable fraction of VWF is secreted immediately after stimulation and released into the medium, while a second fraction of VWF remains at the cell surface for a longer period of time after the actual WPB-PM fusion. The latter process is accompanied by the formation of actin rings or coats at the fused WPB that possibly support the expulsion of highly multimeric VWF [23, 24, 38]. Since both, MyoVc and Spire1 are actin-binding proteins and Spire1 can act as a nucleation factor promoting actin polymerization, we analyzed whether they participate in the regulation of the actin rearrangements at fused WPB and thereby affect the surface presentation of multimeric VWF that requires actin rings for full expulsion [24]. Therefore, we employed an anti-VWF antibody capture assay in which the amount of VWF presented on the cell surface is quantified by binding to anti-VWF antibodies present in the medium [39]. Briefly, confluent HUVEC monolayers transfected with un-targeting siRNA or siRNA directed against MyoVc or Spire1 were incubated with anti-VWF-Dylight650-conjugated antibodies either with or without the addition of histamine. Cells were harvested, stained for total VWF to correct for overall different VWF content between samples, and analyzed via flow cytometry. Note that the antibody capture itself affects the VWF release into the medium by trapping the main bulk of released VWF at the cell surface. The results of these assays are summarized in Fig. 5C, D. While the overall number of cells reacting to histamine via VWF surface presentation is slightly decreased following MyoVc and Spire1 knockdown (Fig. 5C), the median VWF signal intensity at the surface is significantly reduced in both cases (Fig. 5D). This decreased VWF surface presentation after histamine stimulation suggests that the subset of WPB exocytotic events requiring actin assembly for full VWF presentation at the surface is affected by Spire1 and MyoVc depletion.

### MyoVc and Spire1 promote the formation of actin rings at WPB-PM fusion sites

**Fig. 6:**
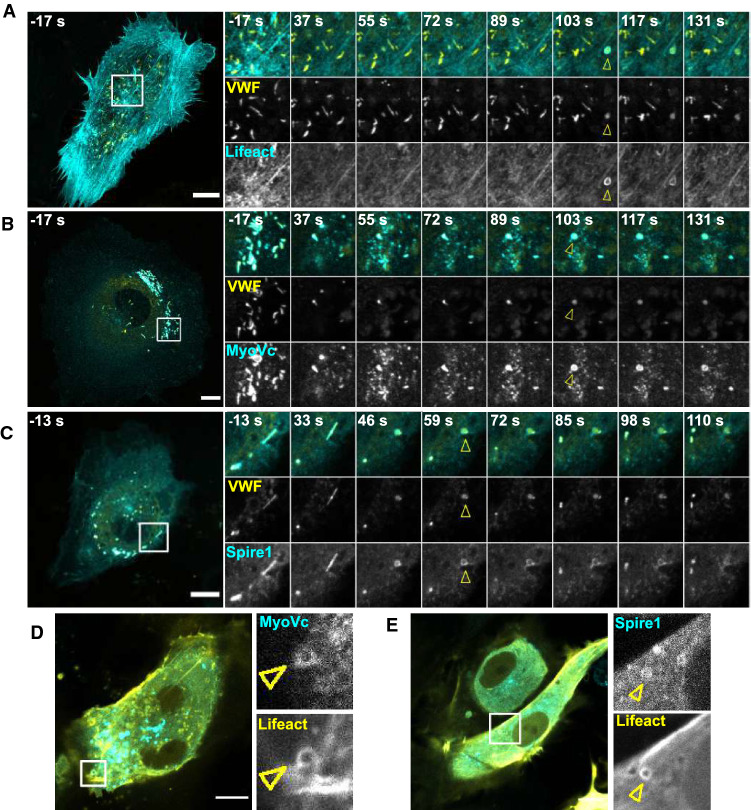
MyoVc and Spire1 localize to actin rings at WPB-PM fusion sites after histamine-evoked exocytosis. Live-cell imaging of transfected HUVEC. **A**–**C** 24 h prior to the recordings, cells were transfected with VWF-RFP (yellow) and GFP-tagged Lifeact (**A**), MyoVc (**B**) or Spire1 (**C**) depicted in cyan. At time point 0 s, HUVEC were stimulated with 500 µM histamine. Shown are still images of maximum intensity projections of *z*-stacks, and time points relative to stimulation are indicated. Detailed views of the regions of interest indicated by boxes in the whole-cell overview (left) are displayed on the right. As shown before by anti-VWF antibody capture analysis [23] individual WPB-PM fusion events can identified by a collapse of the elongated VWF-RFP signal into a round fusion spot that is triggered by neutralization of the acidic luminal pH of WPB. Fusions are non-synchronous and typically occur within 2–3 min after histamine stimulation positive for Lifeact, MyoVc and Spire1, respectively. Yellow arrows indicate fusions accompanied by ring-like structures. **D**, **E** HUVEC were transfected with MyoVc-GFP (**D**) or Spire1-GFP (**E**) (cyan) and Lifeact-mCherry (yellow). Images were taken 2–3 min after stimulation with 500 µM histamine. Shown are stills of single confocal planes. Regions of interest are marked by boxes in the whole-cell merge pictures (left) and single channels are shown enlarged on the right. Yellow arrows indicate ring-like structures. Scale bars: 10 µm

Because the VWF secretion assays suggested an involvement of MyoVc and Spire1 in long-lasting fusion events that are linked to the formation of actin rings at the fused WPB [23], we employed live-cell imaging to track the dynamic behavior of either Lifeact-GFP, MyoVc-GFP or Spire1-GFP after inducing WPB exocytosis with histamine (Fig. 6A–C). This revealed that both, MyoVc and Spire1 accumulate in ring-like structures at WPB fusion sites after addition of histamine. The ring-like structures were remarkably similar to the post-fusion actin rings that had been described before [23, 24] as also visualized in Fig. 6C via expression of Lifeact-GFP. Moreover, in cells ectopically expressing both Lifeact-mCherry and either MyoVc-GFP or Spire1-GFP, ring-like structures are co-labeled for both, actin and Spire1 or MyoVc (Fig. 6D, E).

**Fig. 7:**
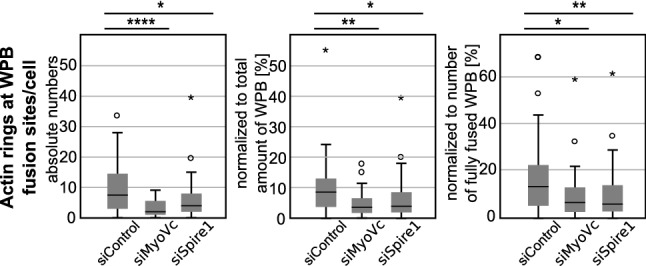
Depletion of either MyoVc or Spire1 reduces the number of actin rings at WPB fusion sites. HUVEC were transfected with siRNA (Control, MyoVc- or Spire1-targeting), Lifeact-GFP and VWF-RFP constructs. 24 h later, life-cell imaging was started and cells were stimulated with histamine (500 µM). Live-cell images were then analyzed by determining the number of WPB before and 5 min after stimulation, and by counting the amount of WPB fusion events accompanied by an actin ring. Shown are box-and-whisker plots depicting the absolute number of actin rings, the number of actin rings normalized to the total number of WPB per cell and the number of actin rings normalized to the number of fully fused WPB. siCtrl: *n* = 50 cells. siMyoVc: *n* = 30 cells. siSpire1: *n* = 50 cells. Statistics employed Kruskal–Wallis and Bonferroni-corrected post hoc tests

The formation of actin rings at WPB fusion sites is likely to depend on de-novo actin nucleation [23, 24] and Spire proteins are able to facilitate nucleation of unbranched actin filaments by binding several actin monomers [40, 41]. Therefore, we investigated whether Spire1 and its binding partner MyoVc participate in the formation and/or maintenance of actin rings at WPB fusion sites after histamine stimulation. To achieve this, we induced a knockdown of either MyoVc or Spire1 and expressed VWF-RFP and Lifeact-GFP to analyze individual WPB-PM fusion events and actin rearrangements by live-cell microscopy. We then determined the overall amount of WPB per cell before and after stimulation and quantified the number of WPB that underwent fusion accompanied by the formation of an actin ring. Both knockdowns reduced the amount of actin rings at WPB fusion sites (Fig. 7). In a next step, we also corrected for an initial difference in the number of WPB between the conditions (e.g., less WPB in MyoVc-depleted cells) and normalized the absolute number of actin rings formed to the overall amount of WPB per cell. As MyoVc promotes secretion of soluble VWF as shown by ELISA experiments (Fig. 5A), we also normalized the number of actin rings to the number of fully fused WPB (as calculated by the difference between the number of WPB per cell before and after stimulation). Also in these analyses, Spire1 and MyoVc depletion resulted in a significant decrease in the number of WPB fusion events that were characterized by the formation of a post-fusion actin ring. In combination with their localization to actin rings at WPB fusion sites, this indicates that both proteins are involved in the formation and/or maintenance of such structures.

## Discussion

In 2011, Nightingale et al. reported for the first time the appearance of actin rings or coats appearing at WPB-PM fusion site after phorbol 12-myristate 13-acetate (PMA) induced stimulation of endothelial cells. The exact composition and function of these actin rings during WPB exocytosis are currently debated and appear to be stimulus-dependent. Classical Ca^2+^ evoking stimuli like thrombin and histamine seem to rely less on actin ring formation compared to cAMP inducing agonists, phorbol 12-myristate 13-acetate (PMA) or vascular endothelial growth factor (VEGF) which induce intracellular Ca^2+^ elevation and protein kinase A (PKA) signaling [21–24, 38, 42]. The formation of contractile actomyosin rings at WPB-PM fusion sites could facilitate the expulsion of large cargo such as highly multimeric VWF, but a direct involvement of myosin II contractility could so far only be observed for cAMP-evoking agonists, VEGF and PMA [22, 24]. In line with this, histamine stimulation does not seem to be as efficient in the expulsion of large cargo VWF compared to other agonists [38] and myosin II inhibition does not seem to affect VWF secretion after histamine stimulation [23]. Hence, actin ring formation might represent a regulatory mechanism to facilitate a stronger stimulus-dependent pro-thrombotic response by actively supporting the externalization of large VWF multimers that are highly pro-coagulant. However, the actin ring could also function in stabilizing the fusion pore to facilitate cargo release, lingering kiss exocytosis or compensatory endocytosis [23, 24, 43, 44].

**Fig. 8:**
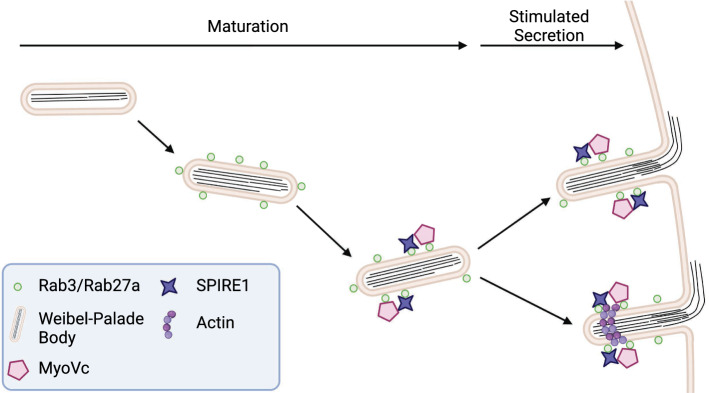
Model illustrating the possible role of MyoVc and Spire1 in WPB exocytosis. During maturation, MyoVc and Spire1 are recruited to WPB via Rab3 and/or Rab27a. Upon secretagogue stimulation WPB undergo exocytosis, with or without the appearance of actin rings. The latter involves the action of Spire1 and MyoVc which are also present in the ring-like structures. Created with BioRender.com

The de-novo formation of rings consisting of polymerized actin at WPB-PM fusion sites suggests the involvement of actin nucleators. Furthermore, Mietkowska et al. reported that Rho signaling is pivotal for the formation of actin rings. Rho signaling in turn might activate Arp2/3, formin or Spire-dependent actin nucleation [45–48]. However, so far no factor that could nucleate actin polymerization at WPB-PM fusion sites had been reported. Via applying proximity proteomics, we identified Spire1 and its interaction partner MyoVc as proteins that are associated with WPB localized Rab GTPases [16]. Here, we could confirm this WPB localization and importantly the dynamic recruitment of both proteins into ring-like structures at WPB fusion sites. As depletion of either protein decreased the amount of surface VWF and the number of actin rings that are formed at WPB fusion sites after histamine simulation, we propose that both proteins play a role in either forming or stabilizing these Ca^2+^-evoked actin structures at WPB fusion sites (Fig. 8). Such putative function of MyoVc differs from the one proposed for myosin IIa in cAMP-evoked VWF secretion where it promotes the formation of a functional actin framework around WPB that facilitates their exocytosis [49]. As an actin nucleator, Spire1 might drive the de-novo formation of the actin ring or might recruit different formins. However, the known interaction partners of Spire1—FMN1 and FMN2 [30, 50, 51]—appear not to be expressed in HUVEC in significant amounts at the RNA (Fig. S7) and protein level [16, 52]. On the other hand, in invadosomes Spire1 seems to form a complex with mDia1 [34] and the human diaphanous homolog 1 was found to significantly associate with both Rab27a and Rab3b in HUVEC (*p* < 0.05) [16].

We show that MyoVc and Spire1 can interact with each other. In accordance with this, all Spire proteins contain a MyoV-binding sequence termed the globular tail-binding motif (GTBM). The corresponding binding sequence in MyoV is conserved and found in the globular tail domain (GTD) of all three MyoV isoforms [26]. Binding of MyoV to Rab GTPases is facilitated via the coiled coil region or the GTD at a site distinct from the Spire-binding site, implicating that MyoV proteins can bind Rab GTPases and Spire proteins simultaneously [26, 35]. While MyoV proteins have been shown to directly interact with several Rab GTPases [29, 53, 54], this is not the case for Rab27a. Instead, for MyoVa an indirect association with Rab27a is mediated by other Rab-binding proteins, e.g., melanophilin [55, 56] or MyRIP which facilitates recruitment to WPB [9]. For MyoVc, complex formation with WPB-bound Spire1/Rab27a and/or the interaction with Rab3 seems to drive its association with WPB. This might be of special interest since so far no direct Rab3 effector interaction has been linked to WPB biology.

Notably, mutation of the Rab3-binding site in MyoVc did not fully abolish recruitment of MyoVc to WPB (Fig. 2D). This could indicate that additional modes of recruitment are in place, e.g., via Spire1 interaction, and could suggest multiple functions of MyoVc in WPB biology. Spire1 localization to WPB is driven by its tail domain and possibly involves interactions with Rab GTPases. Both Rab3 and Rab27 were shown to directly interact with Spire1 [27, 34] and the SB domain of Spire1 alone is sufficient for WPB association, although recruitment seems to be less efficient. This is in accordance with the findings of Alzahofi et al. who reported a strong interaction of Rab27a with Spire1-SB-FYVE-H1 and a reduced but notable interaction with FYVE only and SB-FYVE domains, indicating that the C-terminal domains of Spire1 work in concert to facilitate direct binding to Rab27a [27]. Thus, multiple interactions with different Rab GTPases and with one another likely are responsible for the association of Spire1 and MyoVc with WPB. How the WPB-associated Spire1 and MyoVc are activated following secretagogue-evoked WPB-PM fusion to assist the post-fusion establishment of actin rings remains to be determined.

## Materials and methods

### Cell culture and transfection

HUVEC were acquired from PromoCell as cryo-conserved pools (C-12203) and cultured on Corning CellBind dishes at 37 °C and 5% CO_2_ in 1:1 mixed medium comprising M199 medium (BioChrom) supplemented with 10% FCS, 20 µg/ml gentamycin, 15 µg/ml amphotericin B, and ECGMII (PromoCell) supplemented with 20 µg/ml gentamycin, 15 µg/ml amphotericin B. Experiments were conducted with HUVEC passage 3–5.

HUVEC were transfected using the Amaxa nucleofection system (Lonza) according to the manufacturer’s specifications. Program U-001 was employed with the following modifications: Per cuvette 20 cm^2^ confluent cells and 1–6 µg plasmid DNA or 400–500 pmol siRNA were re-suspended in self-made transfection buffer (4 mM KCl, 10 mM MgCl_2_, 10 mM sodium succinate, 100 mM NaH_2_PO_4_, pH 7.4 adjusted with NaOH or HCl).

### Plasmids and siRNA

Spire1, VWF and Lifeact plasmids have been described previously [6, 23, 27, 57]. MyoVcTail-GFP was cloned by amplification of MyoVcTail (2549 bp) from a cDNA template (Dharmacon, MHS6278-202759992) using the following primers:

- Forward: AGTCTCGAGCGAACAAAGAAAACCATGGGCTGGT
- Reverse: GGTGGATCCCTGCTATAACCTATTCAGAAAGCCTA

The PCR fragment was inserted into the pEGFP-C1 backbone digested with XhoI and BamHI restriction enzymes. MyoVc full-length plasmids were acquired by amplifying the MyoVc N-terminus (4069 bp) with the following primers:

- Forward: TACTCGAGACATGGCGGTGGCCGAGCTGTAC
- Reverse: ACGGTCGACATCATTGGCTTTTCCAATTGTCTT

This fragment was first inserted into the pEGFP-C1 backbone using XhoI and SalI restriction sites and afterward cloned into the MyoVcTail-GFP vector using XhoI and SwaI restriction enzymes.

The MyoVc-GTD (1386–1742) fragment was amplified using the primers given below, and the PCR product (1071 bp) was inserted into an empty pEGFP-C1 using XhoI and BamHI restriction sites:

- Forward: TAGCTCGAGACGCCAAGCTCATTCAGAACCTC
- Reverse: GGTGGATCCCTGCTATAACCTATTCAGAAAGCCTA

The MyoVc-GTD* Rab3-binding-deficient mutant was generated with the Q5^®^ Site-Directed Mutagenesis Kit (NEB) in four consecutive steps using the following primers:

- Forward (1): CCCGGGCTGCCGGCTCATATCCTGTTCATGTGTGTGCGCTA
- Reverse (1): GATCATGCCCACCACGCCCTGGGGCTTCAAGTCAAGAATG
- Forward (2): CACCATGACCGCCGTCCTGCA
- Reverse (2): TAGCCGTCCGTGTCGTCTATG
- Forward (3): CACCATGTGCGACAACGGCCT
- Reverse (3): GTGTAAAAGTAGCTCAGCTG
- Forward (4): TGGCTTAAAGGTAAGAACTTG
- Reverse (4): TTCTTCTAAGTAGCTGATATT

Introduced were the depletion of V1387, the mutation of N1390 (to G, AAC to GGC), R1385 (to Q, CGT to CAG), Q1564 (to D, CAG to GAC) and S1556 (to N, AGC to AAC).

Materials for cloning including restriction enzymes, Q5 polymerase and T4 ligase were purchased from NEB and used according to the manufacturer’s instructions.

siRNAs were obtained from siTools (55930—MYO5C, 56907—SPIRE1). AllStars Negative Control siRNA was purchased from Qiagen (102781).

### Antibodies

Anti-VWF antibodies were acquired from DAKO (mouse monoclonal and rabbit polyclonal). Conjugated anti-VWF antibodies were generated using DyLight antibody labeling kits 405 or 650 (ThermoScientific, 53020 or 84535) and polyclonal rabbit anti-VWF antibodies (DAKO) according to the manufacturer’s instructions. Anti-MyoVc (32190002) and anti-calnexin (610524) antibodies were obtained from Novus Biological or BD Transduction Laboratories, respectively. Secondary antibodies (AlexaFluor488, AlexaFluor568, AlexFluor647) were purchased from Molecular Probes.

### Western blot analysis

For preparation of cell lysates, HUVEC were harvested using trypsin/EDTA. After washing once with PBS, cell pellets were re-suspended in 30 µl RIPA buffer containing protease inhibitors (EDTA-free protease inhibitor cocktail, Roche) per 20 cm^2^ confluent HUVEC and lysed for 30 min on ice followed by 1 min sonification. Cellular debris was removed by centrifugation at 10,000×*g* for 10 min at 4 °C. Protein concentrations were determined using the Pierce 660 nm protein assay (ThermoFisher). Samples were diluted 1 to 6 with 6 × protein loading buffer and incubated for 10 min at 95 °C. Buffers were set up according to Hung et al. [58].

Samples were subjected to 8% SDS-PAGE for 30 min at 70 V and subsequently at 120 V, and blotted onto 0.2 µm nitrocellulose membrane in a wet tank system at 115 V for 1 h at 4 °C in Tris–Glycine buffer (25 mM Tris, 190 mM glycine, 20% (v/v) methanol). Membranes were blocked using 5% skim milk in TBST (150 mM NaCl, 50 mM Tris–HCl, 0.1% Tween 20, pH 7.6) for at least 30 min and incubated with primary antibodies over night at 4 °C. For signal detection, infrared conjugated secondary antibodies (IRdye680RD or IRdye800CW, LICOR) and the Odyssey imaging system (LICOR) were used.

### Real-time quantitative PCR

Cells were harvested and total RNA was extracted using TRIzol (Invitrogen) according to the manufacturer’s instruction. 1 μg was transcribed into cDNA using the High‐Capacity cDNA Reverse Transcription Kit with random primers (ThermoFisher). qPCR was conducted using Brilliant III SYBR green qPCR mastermix (Argilent technologies) and primers directed against Spire1 (Microsynth, forward: CCAGTGGAATGCCTCGCTCTT, reverse: GAAAACCTCCTGGTTCGGCAA), MyoVa (Qiagen, QuantiTect Primer Assays, Hs_MYO5A_1_SG), MyoVb (Microsynth, forward: AACGTGGGCATGGAGAACAAGG, reverse TTCTTCAGCCGCTCTACCTCCA), MyoVc (Microsynth, forward: TACAGCCGAGGATTCCTGGCAA, reverse: GCACGAATCGTCGGATACTCTG), FMN1 (Microsynth, forward: CATGAGAAGGAGTCGCTAAGAGC, reverse: TGAAGTCTGCCAGGAGTCCTGT), FMN2 (Microsynth, forward: GTTTCCTAGGCGAGTTCCATCC, reverse: CTTCTGGACAGCATCTGAGCGT) and housekeeping gene b‐2 microglobulin (Microsynth, forward: CCACTGAAAAAGATGAGTATGCCT, reverse: CCAATCCAAATGCGGCATCTTCA) on a CFX 384 Real‐Time PCR Cycler. The analysis of qPCR results is described in detail in Schloer et al. [59].

### ELISA-based VWF secretion assay

Transfected HUVEC were seeded on collagen-coated 24-well plates. Cellular debris was removed by washing twice with phosphate-buffered saline (PBS) 3–4 h post transfection. The following day, cells were incubated in 200 µl of starvation medium (M199 + 1% BSA) per well. After 20 min, 150 µl of the supernatant per well was removed carefully and diluted in 400 µl blocking buffer (1.5% BSA, 0.1% Tween 20 in PBS) (basal secretion). Remaining medium was aspirated and cells were incubated in 200 µl starvation medium containing 100 µM histamine (Sigma-Aldrich) for 20 min. Per well, 100 µl of the supernatant was removed and diluted in 400 µl blocking buffer (stimulated secretion). Remaining medium was aspirated and cells were lysed by 30 min incubation on ice in 200 µl cold starvation medium containing 0.1% Triton X-100 and protease inhibitors (EDTA-free protease inhibitor cocktail, Roche). 50 µl of this cell lysate was diluted in 800 µl blocking buffer (residual). High-binding 96-well ELISA plates (Greiner Bio One) were prepared by O/N incubation at 4 °C with 200 µl coating buffer (50 mM carbonate, pH 9.6) containing anti-VWF antibodies (polyclonal rabbit, DAKO, diluted 1:1000) per well. Wells were washed (0.1% Tween 20 in PBS) and unspecific antibody binding was reduced by addition of blocking buffer for 2 h. The blocking buffer was aspirated, and to each well, 100 µl of diluted samples (basal or stimulated or residual) was added. Samples were incubated O/N at 4 °C and subsequently washed with 0.1% Tween 20 in PBS. Anti-VWF conjugated to HRP (DAKO) was diluted in blocking buffer (1:8000) and added to the 96-well plate. After 2 h at RT, samples were extensively washed and TMB Substrate (Pierce) was added. After 10 min the reaction was stopped by addition of 2 M sulfuric acid and the amount of reaction product was determined photometrically at 450 nm. Each condition was analyzed in triplicates (3 × 24-well per condition) and each sample (basal, stimulated or residual) was analyzed in quadruplicates.

### Flow cytometry-based VWF secretion assay

Transfected HUVEC were seeded on collagen-coated 12-well plates. Cellular debris was removed by washing twice with phosphate-buffered saline (PBS) 3 to 4 h post transfection. The following day, cells were incubated in starvation medium containing polyclonal rabbit anti-VWF antibodies (1:400) for 20 min to block unspecific binding sites, followed by another 20 min incubation time with DyLight650-conjugated anti-VWF antibodies (1:400 in starvation medium) to label acutely externalized VWF. Per condition one well contained in addition 100 µM histamine. Subsequently, cells were washed with PBS and carefully harvested using Accutase (Millipore). Cells were pelleted (4 min, 200×*g*), washed again in PBS and fixed using the BD Cytofix/Cytoperm™ solution. Afterward, cells were blocked in 2% BSA in PBS and stained for total VWF content by incubation with anti-VWF antibodies conjugated to DyLight405 (1:2000, 1 h) in BD Perm/Wash™ solution. Cells were then subjected to flow cytometric analysis. Per condition 5000 cells were counted.

### Pulldown via GFP-Trap

Pull-down experiments were conducted using ChromoTek Agarose GFP-Trap^®^ beads according to the manufacturer’s protocol with the following change: instead of starting from one 10 cm dish per condition, we increased the amount of starting material by lysing three 10 cm plates per condition, but still used 25 µl of beads.

### Immunofluorescence stainings

Cells were seeded on collagen-coated coverslips (12 mm diameter) until they reached 80% confluency, then fixed in 4% PFA in PBS for 10 min at RT and permeabilized using 0.1% Triton X-100 in PBS for 4 min. Unspecific binding was blocked by addition of 3% BSA in PBS for at least 30 min, followed by antibody incubation O/N at 4 °C (1:200 dilution, except for anti-VWF antibodies that were diluted 1:800) in 3% BSA in PBS. Secondary antibodies were diluted 1:200 and incubated for 40 min at RT. After extensive washing, samples were mounted in mounting medium.

### Microscopy

Confocal microscopy was performed using LSM 800 or LSM 780 microscopes (Carl Zeiss) equipped with a Plan-Apochromat 63 ×/1.4 oil immersion objective. Life-cell imaging was conducted at 37 °C in medium containing 20 mM HEPES on 8-chamber µ-slides (Ibidi). Cells were stimulated by addition of 500 µM histamine.

### Image analysis

Confocal images were analyzed using ImageJ. For determining the number of WPB and the WPB distance to nucleus, maximum intensity projections of z-stacks were used and regions of interest (ROI) were manually selected to outline the borders of individual cells. XM and YM coordinates of the nucleus were determined within the DAPI channel. The VWF channel was thresholded and particles with a size greater than 0.1 µm^2^ were counted. Individual XM and YM coordinates were determined to calculate the distance of each WPB to the nucleus.

### Statistics

All statistics were performed using IBM SPSS Statistics. Asterisks mark statistically significant results: ****p ≤ 0.0001, ***p ≤ 0.001, **p ≤ 0.01, *p ≤ 0.05. Normal distribution was assessed by the Shapiro–Wilk Test, *p* < 0.05. Normally distributed data were analyzed employing Student’s *t* test or one-way ANOVA and Bonferroni correction. Non-parametric data were analyzed using Kruskal–Wallis and Bonferroni correction.

### Supplementary Information

Below is the link to the electronic supplementary material.

Supplementary file1 (PDF 496 KB)

## Declarations

### Data availability

The datasets generated during and/or analyzed during the current study are available from the corresponding author (gerke@uni-muenster.de) on reasonable request.
