# Supplementary material for: Spire1 and Myosin Vc promote Ca2+-evoked externalization of von Willebrand factor in endothelial cells
Source: Cell Mol Life Sci. 2022 Jan 27;79(2):96. doi: 10.1007/s00018-021-04108-x (PMC8794916; doi:10.1007/s00018-021-04108-x)
Supplement: Supplementary file 1 — Supplementary file1 (PDF 496 KB) [file 18_2021_4108_MOESM1_ESM.pdf]

Supplemental information

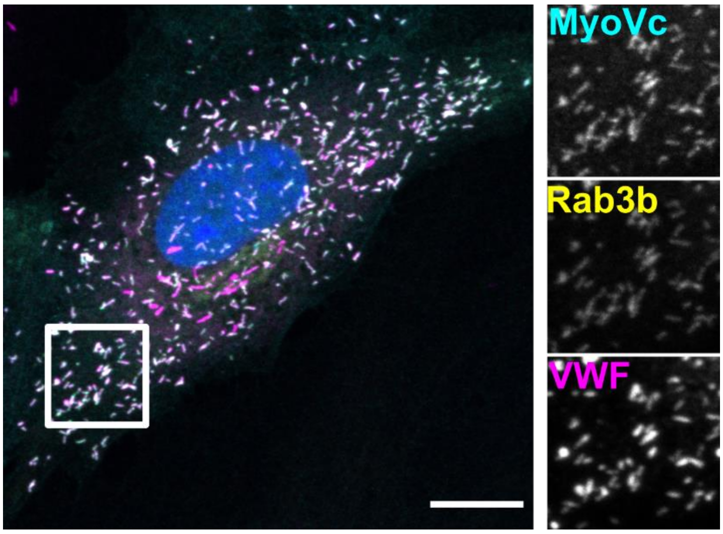

Figure S1: **MyoVc-GTD colocalizes with Rab3b on WPBs.** HUVEC were transfected with Rab3b-mApple (yellow) and MyoVc-GTD-GFP (cyan) and stained for VWF as general WPB marker (magenta) after fixation. DAPI staining is shown in blue. Boxed peripheral region is enlarged on the right side. Scale bar: 10 µm.

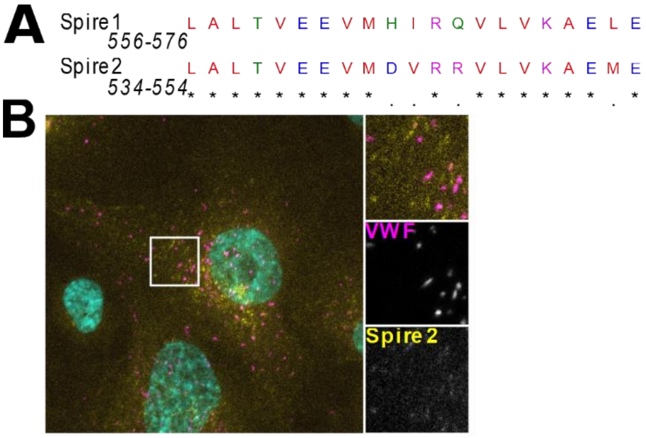

Figure S2: **Spire2 does not localize to WPB. A.** Amino acid sequence alignment of the Spire Box (SB) domains of Spire1 and 2. **B.** Ectopic expression of Spire2-GFP (yellow) in HUVEC. Cells were stained for the WPB marker VWF (magenta). DAPI stainings are shown in cyan. Boxed area is shown enlarged on the right side.

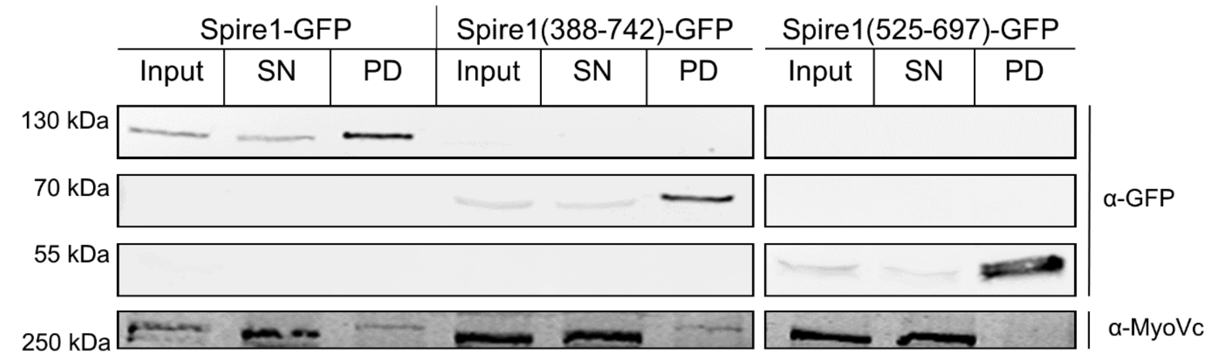

Figure S3: **Spire1 interacts with MyoVc via a region harboring the GTBM or the H2 domain.** HUVEC were transfected with Spire1-GFP or different GFP tagged Spire1 truncation mutants namely Spire1 amino acid (aa) 388-742 (GTBM-SB-FYVE-H2, Spire1Tail) and Spire1 aa 525-697 (SB-FYVE). Cell lysates were directly analyzed by Western blot (Input) or further processed using GFP-trap pulldowns. GFP-trap bound proteins (pulldown, PD) and the supernatant (SN) of the GFP-trap samples were also analyzed by Western blots using antibodies directed against GFP or MyoVc as indicated.

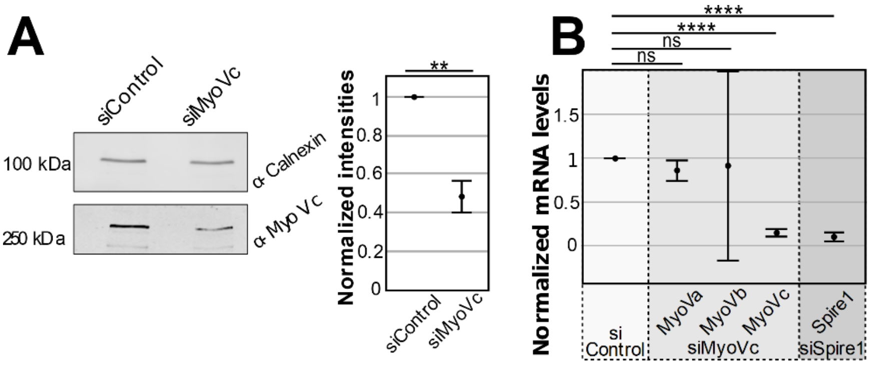

Figure S4: **siRNA mediated knockdown of MyoVc and Spire1. A** HUVEC were transfected with untargeting or MyoVc targeting siRNA. Transfection was repeated 48 h later. The following day, cells were lysed and analyzed via Western Blot. Calnexin was used as a loading control. MyoVc band intensities were measured and normalized to intensities of the loading control. n=5. Statistics employed Student´s t-test. **B** HUVEC were transfected with untargeting (siControl), MyoVc or Spire1 directed siRNA. Transfection was repeated 48 h later. The following day, RNA was isolated, transcribed into cDNA and analyzed by quantitative PCR using primers directed against MyoVa, MyoVb, MyoVc or Spire1 and b-2 microglobulin (housekeeping gene). Note that Ct values for MyoVb primers were 33.96 ± 0.93 (siCtrl) and 34.43 ± 1.79 (siMyoVc) indicating very low expression levels. Gene expression was analyzed using the ΔΔCt method. n=4. Statistics were conducted using the Student´s t-test on ΔCt values. Error bars show standard deviations. ****$p \leq 0.0001$, ***$p \leq 0.001$, **$p \leq 0.01$, *$p \leq 0.05$.

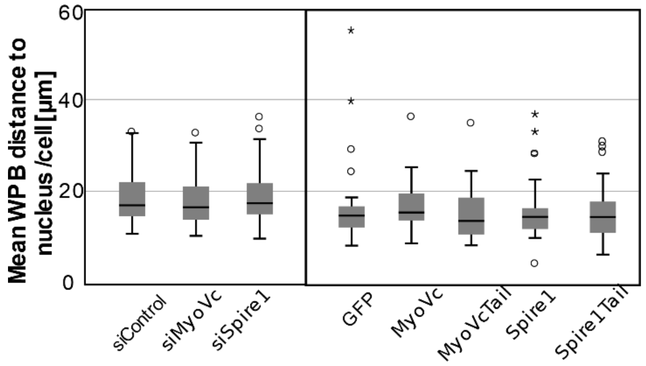

Figure S5: **MyoVc and Spire1 do not affect the peripheral distribution of WPB in a significant manner.** HUVEC were transfected with siRNA (Control, MyoVc- or Spire1-targeting) or empty GFP, MyoVc or Spire1 constructs. Cells were fixed and immunostained for WPB markers. Confocal sections were imaged and analyzed for intracellular WPB distribution as described in Materials and Methods. Shown are box-and-whisker plots depicting the mean WPB distance to nucleus per cell. siCtrl: n=56 cells. siMyoVc: n=64 cells. siSpire1: n=57 cells. GFP: n=18 cells. MyoVc: n=15 cells. MyoVcTail: n=28 cells. Spire1: n=23 cells. Spire1Tail: n=26. Statistics employed Kruskal-Wallis and Bonferroni corrected post hoc tests.

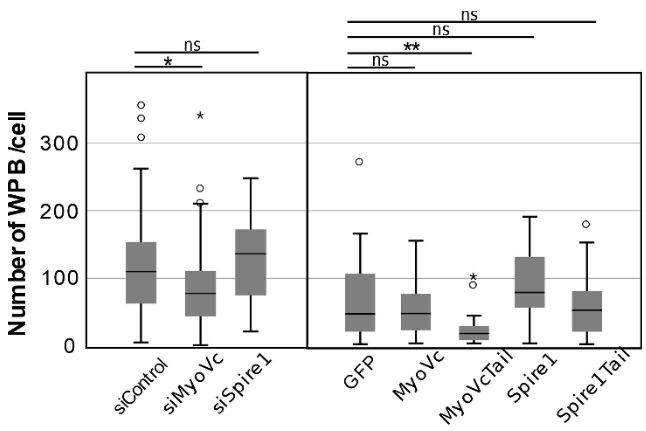

Figure S6: **MyoVc affects the number of WPB per cell.** HUVEC were transfected with siRNA (Control, MyoVc- or Spire1-targeting) or empty GFP, MyoVc or Spire1 full length and mutant (tail) constructs. Cells were fixed and immunostained for WPB markers. Confocal sections were imaged and analyzed as described in Materials and Methods. Shown are box-and-whisker plots depicting the mean number of WPB per cell. siCtrl: n=56 cells. siMyoVc: n=64 cells. siSpire1: n=57 cells. GFP: n=18 cells. MyoVc: n=15 cells. MyoVcTail: n=28 cells. Spire1: n=23 cells. Spire1Tail: n=26. Statistics employed Kruskal-Wallis and Bonferroni corrected post hoc tests. ****$p \leq 0.0001$, ***$p \leq 0.001$, **$p \leq 0.01$, *$p \leq 0.05$.

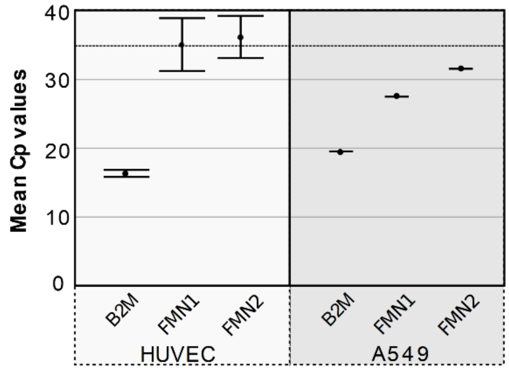

Figure S7: **HUVEC do not seem to express FMN1 or FMN2.** HUVEC mRNA was isolated, transcribed into cDNA and analyzed via qPCR with primers directed against B2M (housekeeping gene), FMN1 or FMN2. Shown are mean Cp values (± SD). Cp values above 35 were considered a cutoff for expression. Primer melting curves for all three primers showed only one peak indicating specificity. n=4. To validate the functionality of the primer pairs used, cDNA from A549 cells that are known to express both FMN1 and FMN2 was analyzed. n=1.
